# Knowledge and first aid management of choking children among parents in a tertiary care hospital, Sri Lanka

**DOI:** 10.1186/s12873-025-01450-2

**Published:** 2025-12-18

**Authors:** Nammuni Kushmitha Keshara Thabrew, Shasanthi Lakshika Udawaththa, Ransi Nimesha Thenuwara, Savindi Chamathika Tissera, Erandi Uthpala Siriwardhana, Subangi Sivaganeshan, Deegoda Gamage Dewni Tharushika, Heseetha Thananchayan, Majury Thirugnanaselvan, Nisal Dulanjith Sooriyaarachchi, Shanmugaratnam Sivakumar, Samath Dhamminda Dharmaratne, Lahiru Sandaruwan Galgamuwa

**Affiliations:** 1 Faculty of Medicine, University of Peradeniya, Peradeniya, Sri Lanka; 2 Department of Community Medicine, Faculty of Medicine, University of Peradeniya, Peradeniya, Sri Lanka; 3 Department of Parasitology, Faculty of Medicine, Sabaragamuwa University, Belihuloya, Sri Lanka

**Keywords:** Choking, First aid, Knowledge, Parents

## Abstract

**Background:**

Choking is a critical medical emergency caused by the obstruction of airway due to foreign objects. Timely and appropriate first aid is essential to prevent further complications and save the lives of choking victims, especially children. However, inadequate knowledge of first aid management in choking can lead to delayed or incorrect interventions, resulting in significant morbidity and mortality among children. Therefore, this study aimed to determine the level of knowledge and identify the sources of information regarding first aid management of choking among parents whose children were admitted to a Specialized Children Hospital, Sri Lanka.

**Methods:**

A descriptive cross-sectional study was carried out including 425 parents of children admitted to Sirimavo Bandaranayake Specialized Children Hospital, Peradeniya, Sri Lanka. An interviewer-administered questionnaire was used to collect data on socio demographic characteristics, awareness of potential choking hazards and first aid management. Collected data were analyzed using SPSS software. The knowledge was categorized as good or poor using a 50% cut-off value.

**Results:**

Majority of caregivers were mothers (*n* = 343, 80.7%). Overall, 38.8% of participants demonstrated good knowledge regarding choking first aid with the mean knowledge score (12.8 ± 3.95). Although general awareness of choking first aid was high (87.8%), only 18.8% had received prior first aid training. The main sources of information included healthcare professionals (53.1%), media (46.9%), and family members (41.6%). Notably, 21.9% correctly identified universal sign of choking as clutching the throat, and 50.8% recognized the symptoms of complete airway obstruction. However, only 10.4% were aware that first aid for choking should be initiated within three minutes. However, no significant association was identified between the knowledge of first aid for choking children with first aid training, male gender, age and educational attainment of their parents.

**Conclusions:**

Parental knowledge regarding the first-aid management of choking is insufficient. Parents with prior first-aid training demonstrated better knowledge. Implementing parental education programs and comprehensive first aid training would enhance knowledge and skills in managing choking incidents and reducing childhood morbidity and mortality.

## Introduction

The term “choking” refers to the physical obstruction of the airway by food or other objects, which interferes with normal breathing [1]. It is a medical emergency that requires immediate action by bystanders to prevent serious complications or death [1]. Choking remains a major cause of preventable injury and death, especially in children, due to its potentially fatal outcomes. The risk is significantly higher at both ends of the age spectrum, with young children being particularly vulnerable [2]. This increased risk is mainly due to their developing anatomy and physiology, as well as typical childhood behaviors.

Children are physiologically predisposed to foreign body aspirations due to an underdeveloped and inefficient cough reflex, which generates insufficient expiratory force to effectively clear airway obstructions. Additionally, their anatomically narrower airways increase susceptibility to occlusion by aspirated materials and subsequent bronchospasm following obstruction [3]. From a behavioral perspective, young children often lack awareness of choking hazards and have a natural tendency to place objects in their mouths, elevating the risk of airway compromise. Other contributing factors include delayed dentition, poor masticatory efficiency, ingestion of developmentally inappropriate foods, eating while distracted, and underlying neuromuscular or structural disorders affecting swallowing and chewing. Collectively, these factors substantially increase the incidence of foreign body aspiration in this vulnerable age group [4, 5].

Choking most commonly occurs during eating or drinking and is often associated with impairments in neuromuscular coordination or central nervous system function. It may result from aspiration of food or liquids including breast milk in infants as well as inhalation or ingestion of foreign bodies, aspiration of vomitus in patients with altered consciousness, or oral exploration behaviors seen in infants, particularly during teething. Frequently aspirated non-food items include coins, small toys, balloons, marbles, and pen caps. In some cases, certain medications, when improperly ingested, may also lead to airway obstruction [4].

Rapid and effective first aid intervention is critical in preventing severe complications. Airway obstruction lasting more than three to five minutes may result in loss of consciousness, with progression to cerebral hypoxia and potential fatality within minutes if not promptly addressed [6]. The brain, despite comprising only 2% of body weight, consumes approximately 20% of the body’s oxygen supply and is exquisitely sensitive to oxygen deprivation, with cellular injury beginning within minutes and irreversible neuronal damage occurring after approximately 4–6 min of complete oxygen deprivation. Hypoxic-ischemic brain injury is the leading cause of death in pediatric choking cases, surpassing other complications such as pulmonary hemorrhage [7].

Vulnerable brain regions with high metabolic demands—including pyramidal neurons of the hippocampus (CA1 region), cerebral cortex (layers 3, 5, and 6), basal ganglia (caudate nucleus and putamen), and Purkinje cells of the cerebellum—are selectively damaged, resulting in laminar necrosis and permanent neurological deficits. Clinical outcomes in pediatric choking victims who develop hypoxic brain injury include prolonged hospitalization, recurrent pneumonia, severe neurological disability requiring extensive rehabilitation, and frequently death, even when initial resuscitation is successful [7]. The severity of neurological sequelae correlates directly with the duration of hypoxia, emphasizing that immediate intervention by parents or caregivers present at the scene is critical—not merely beneficial—for survival and functional recovery. Unlike other medical emergencies where delayed treatment may result in suboptimal outcomes, delayed intervention in choking emergencies invariably results in catastrophic and irreversible brain damage, making parental knowledge and competence in choking management an absolute prerequisite for child safety [8].

The standard first aid approach for choking begins with scene assessment and severity determination. For mild airway obstruction where the child can cough forcefully, speak, or breathe, should be encouraged to cough spontaneously to expel the object, with medical assistance sought if the obstruction persists beyond a few minutes. Severe obstruction, confirmed by asking “Are you choking?” and observing inability to cough forcefully, speak, or breathe, requires immediate intervention with a combination of back blows and abdominal thrusts. The rescuer should support the child and deliver up to five forceful blows between the shoulder blades using the heel of the hand. If unsuccessful, the rescuer positions behind the child, places a fist on the abdomen at the umbilicus level aligned with the hip bones, grasps the fist with the other hand, and delivers five forceful inward and upward abdominal thrusts (Heimlich maneuver). This cycle of five back blows followed by five abdominal thrusts should be repeated until the object is dislodged or the child loses consciousness. If unconsciousness occurs, the child should be lowered to the ground, emergency medical services activated, and an automated external defibrillator (AED) obtained if available. Cardiopulmonary resuscitation (CPR) should commence immediately with chest compressions. After the first 30 compressions, the mouth should be inspected and any visible foreign object removed before attempting two rescue breaths. If ventilation remains unsuccessful, the cycle continues with chest compressions and mouth inspection before each ventilation attempt, as compressions may generate sufficient intrathoracic pressure to expel the obstruction [9].

Parents were deliberately selected as the study population because they are the most critical first responders in pediatric choking emergencies. The ability to recognize and manage choking episodes is particularly vital for parents and caregivers of children aged 0–10 years, who are at elevated risk due to developmental and behavioral factors. Parental engagement significantly influences both the prevention and outcome of injury events within the home environment [10]. Despite adult supervision, a substantial proportion of pediatric choking incidents continue to occur, highlighting a critical gap in caregiver awareness and effective monitoring. Moreover, hesitation to intervene is frequently reported among bystanders, often attributed to inadequate knowledge or lack of confidence in first aid procedures [1]. Data regarding Sri Lankan parents’ knowledge of pediatric choking and appropriate first aid responses remain scarce. Therefore, the present study aimed to assess parental awareness and understanding of first aid management for choking, in a Specialized Children’s Hospital in Sri Lanka.

## Methodology

This study designed to assess the knowledge on first aid management of choking among parents of children admitted to Sirimavo Bandaranayake Specialized Children’s Hospital, Peradeniya, Sri Lanka.

A descriptive cross-sectional study was conducted in the SBSCH from October 2022 to December 2022. Parents of children aged within 0–10 years and were admitted to this hospital were included for this study. Parents who are medical doctors or nurses were excluded because they have received prior training on choking first aid during their clinical training and would therefore not represent the knowledge of general population.

Sample size was calculated using the following equation n = Z^2^_1−α/2_ p (1-p)/d^2^, where n = sample size, p = anticipated population proportion (0.5), Z1-α/2*2 = confidence interval (95%, 1.96) and d = precision (0.05) [*n* = 1.96^2^ × 0.5(1-0.5)/0.05^2^]. The calculated sample size was 384 and to accommodate for non-respondents an additional 10% was added to the calculated sample size. Therefore, the final sample size was 422. Data was collected using an interviewer-administered questionnaire which had questions regarding socio demographic factors and knowledge of choking first aid.

Subjects were selected using the consecutive sampling technique, where every subject that meets the inclusion criteria was included in the sample until the required sample size was achieved. Of the 452 parents approached, 425 consented to participate in the study (response rate: 94%, refusal rate: 6%). Given the Sri Lankan community’s supportive attitude toward educational research, participant recruitment proceeded smoothly. Non-participation (*n* = 27) was primarily attributed to parental fatigue from attending to their hospitalized child, time constraints, and other personal priorities.

The present study used the questionnaire that was used in the study done in Ethiopia with few modifications under the guidance of Consultant Pediatricians working in Sirimavo Bandaranayake Specialized Children’s Hospital, Peradeniya [1]. The questionnaire was initially prepared in English and subsequently translated into Sinhala and Tamil, which are native languages in Sri Lanka. It was then back translated into English to ensure consistency across all versions. This procedure was done by authorized attorney at laws. A pilot testing was carried out prior to data collection with 20 participants in the outpatient department in the Sirimavo Bandaranayake Specialized Children’s Hospital, Peradeniya, Sri Lanka.

The questionnaire consisted of socio demographic information including age, gender, level of education, marital status, and previous first aid training experience. In addition, knowledge of parents regarding first aid for choking was assessed. This included participants’ awareness of sources of first aid information, recognition of the universal sign of choking, anticipated behavior of the victim following choking incidents, factors contributing to choking, potential choking hazards, the critical time period for initiation of first aid intervention, symptoms of airway obstruction, strategies for prevention of choking and knowledge on management of choking incidents giving case scenarios. The prepared questionnaire was tested for validity and reliability during the pilot study. The Cronbach’s alpha values for the knowledge of first aid for choking, potential choking hazards, and knowledge on management of choking incidents were 0.84, 0.82, and 0.90, respectively.

A scoring system was implemented to categorize the level of knowledge by awarding 1 mark for each correct answer and 0 mark for each incorrect answer. The minimum possible score was 0 while the maximum was 28. Parents who achieved a score of 50% and above were considered knowledgeable [11].

### Interviewers training

A training session was conducted for all interviewers by the project supervisor covering the study objectives and questionnaire content, proper question administration techniques, probing techniques for incomplete responses without leading participants, cultural sensitivity and respectful communication with parents and confidentiality and ethical protocols. Data was collected by interviewers from the same ethnic group as the participants, allowing for better communication and clarification. Interviewers trained to ask questions without inflection or emphasis that might suggest a “correct” answer and to avoid nonverbal cues.

Data quality was ensured throughout the research process, from collection through analysis. During the data collection phase, supervisors and the principal investigator conducted daily checks of data collectors’ questionnaires, administration techniques and verified data completeness, providing immediate feedback. Questionnaires were systematically coded during data collection to maintain organization and traceability. Any problems encountered were promptly discussed among the survey team and resolved.

### Statistical analysis

Data was entered into a Microsoft Excel database and analyzed using SPSS version 22. Discrete variables were analyzed with ratios and percentages, while continuous variables were analyzed with means and standard deviations. Chi-square test was conducted to assess the relationship between knowledge status and gender, previous first aid training, level of education and age. Logistic regression was then used to assess the association between socio demographic characteristics and first aid knowledge of choking. Unadjusted and adjusted odds ratios with 95% Confidence intervals and p values were reported to determine significant factors with the knowledge of choking among mothers. The Hosmer-Lemeshow test was used for goodness of fit and calibration for logistic regression models. *p* value less than 0.05 with 95% confidence level was used as significant.

## Results

### Socio-demographic characteristics

A total number of 425 parents with mean age of 34.5 years (age range of 20–48 years) were participated for this study. Majority of the participants were mothers (80.7%), 30–39 age group (60.5%), educated up to ordinary level (50.1%) and did not experience of trained first aid treatments before (81.2%). However, majority of participants (87.8%) heard about first aid provision for a choking child from health professionals (53.1%) followed by electronic media (46.9%), family members (41.6%), and first aid institutions (9.7%) such as St. John’s Ambulance, the Kitty program, Girl Guide associations, the Red Cross Society in Sri Lanka (Table 1).

**Table 1:** Socio-demographic characteristics of the study population

	Variable	Frequency	Percentage
Gender	Male	82	19.3
	Female	343	80.7
Age (Years)	20–29	86	20.2
	30–39	257	60.5
	≥ 40	82	19.3
Level of education	Up to grade 5	20	4.7
	Grade 6–11	213	50.1
	Grade 12–13	165	38.8
	University education	27	6.4
Previous first aid training	Yes	80	18.8
	No	345	81.2
Sources of knowledge on choking first aid	Health professionals	198	53.1
	Media	175	46.9
	Family members	155	41.6
	First aid institution	36	9.7
	Others	51	13.7

## Knowledge of the parents on first aid management of choking children

Out of the total participants, only 21.9% of participants were able to correctly identify the universal sign of choking as clutching the throat. Over half (50.8%) accurately recognized key symptoms of complete airway obstruction, such as the inability to speak or cough (Table 2). Majority of participants (78.8%) correctly acknowledged fluid aspiration as a potential cause of choking. When considering common choking hazards, the majority identified Rambutan (*Nephelium lappaceum*) and Weralu (*Elaeocarpus serratus*) seeds (85.2%) as predominant risks in Sri Lanka followed by coins (74.6%), toffee (61.9%), and chickpeas (32.5%). In addition, majority of participants exhibited awareness of high-risk behaviors in children that contribute to choking, including eating while running, playing, talking, laughing, or inserting objects into the mouth. While 70.4% correctly identified running with food as a significant risk factor, only 14.8% recognized delayed dental development as a potential contributor to choking incidents (Table 2).

**Table 2:** Assessment of knowledge of choking

Question	Correct answer/s	Frequency	Percentage
The universal sign of choking	Clenching at the throat	93	21.9
Symptoms of complete airway obstruction	Inability to produce sound and cough	216	50.8
Symptoms of partial airway obstruction	Noisy breathing and wheezing	2	0.5
Choking induced by aspiration of fluids	Yes	335	78.8
Golden time for initiation of providing choking first aid	3 min	44	10.4
Child’s risk behaviors	Playing	112	26.4
	Running while eating	238	56
	Putting objects in their mouth	254	59.8
	Playing, talking and laughing while eating	317	74.6
Factors leading to choking among children	Improper chewing of food	191	44.9
	Insufficient maturation of teeth	63	14.8
	Running with food in their mouth	299	70.4
	Adventurous nature	134	31.5
Potential choking hazard item	Rambutan, Weralu seeds	362	85.2
	Toffee	263	61.9
	Coins	317	74.6
	Chickpea	138	32.5
Knowledge on choking prevention	Keep small things away from children	330	77.6
	Stop talking while eating	249	58.6
	Proper chewing of food	200	47.1

In terms of preventive knowledge, a total of 77.6% of participants acknowledged the importance of keeping small objects out of children’s reach. Approximately half of participants were aware of the preventive value of discouraging talking while eating and ensuring proper mastication. When considering the critical time frame for initiation of delivering first aid during a choking episode, 74.8% believed it to be within two minutes; however, only 10.4% correctly identified the window as three minutes (Table 2).

In cases of complete airway obstruction without a visible foreign body, a total of 66.4% of participants indicated that they would administer back blows. Clinical guidelines recommend delivering five back blows as the initial intervention for choking children. If this measure fails, the subsequent step involves performing five abdominal thrusts; however, only 4.5% of respondents correctly identified this protocol. Majority of parents (80.9%) expressed readiness to seek medical care promptly.

Despite guidelines advising five back blows, only 27.6% of participants accurately reported this procedure, while 55.6% correctly identified the anatomical site for administering back blows. When faced with a choking child with a visible and accessible foreign object, careful manual removal is advised; this was recognized by 38.1% of respondents. In situations where a choking child develops a cough, 52.9% of participants would still perform back blows, whereas only 19.8% would correctly encourage the child to cough. 23.3% opted to offer water here. In partial airway obstruction, if the cough becomes ineffective, back blows should be administered. If a child becomes unconscious due to choking, maintaining airway patency and attempting to clear the obstruction is critical. Only 22.1% of participants correctly chose to give two rescue breaths and chest compressions. Instead, 27.3% would take the child to the nearest healthcare facility. For infants, 56% correctly identified back blows as the initial management step (Table 3).

**Table 3:** Knowledge of the management of choking

Question	Correct answer/s	Frequency	Percentage
First step in the management of	Back blows	282	66.4
choking children			
If the first attempt failed, the next step is	Five abdominal trusts	19	4.5
Times of back blows should be performed	5 times	113	27.6
Site of the body to provide back blows	Between shoulder blades and base of the ribs	212	55.6
First step of a choking child with visible and assessable foreign body	Remove the foreign body by yourself	162	38.1
If child is choking and coughing	Encourage him to cough and call ambulance	84	19.8
When the child chokes and loss consciousness	Two rescue breaths and chest compressions	94	22.1
The initial step for an infant	Back blows	238	56

Among the participants, 38.8% achieved a score exceeding 14 out of a possible 28 points (greater than 50%) on the knowledge assessment questionnaire. The mean score of this study was 12.8 (45.7%) with a standard deviation of 3.95. Based on these results, approximately 38.8% of participants demonstrated adequate knowledge of choking first aid (Table 4).

**Table 4:** Distribution of scores for the questionnaire among the study sample

Total score	Frequency	Percentage
≥ 50%	165	38.8
< 50%	260	61.2
Total	425	100.0

Notably, 79.1% of respondents reported confidence in their ability to manage choking incidents, while 20.9% self-identified as lacking knowledge in administering first aid for choking children. Among those who expressed confidence, only 43.1% attained a knowledge score exceeding 50%. 

Logistic regression analysis was applied to analyze factors associated with knowledge of choking for first aid. The binary logistic regression showed two significant factors associated with knowledge of first aid management for a choked child: previous first aid training experience (OR = 0.605, 95% CI: 0.370–0.987, *p* = 0.044) was a positive predictor. Males were higher knowledge levels compared to females (OR = 0.602, 95% CI: 0.370–0.978, *p* = 0.041). Other variables like age and education level did not show significant associations in the binary analysis.

However, in multivariate logistic regression, none of these factors remained statistically significant. Gender (adjusted OR = 0.664, 95% CI: 0.394–1.120, *p* = 0.125), previous first aid training (adjusted OR = 0.750, 95% CI: 0.448–1.253, *p* = 0.272), age groups, and educational levels (*p* > 0.05) all showed non-significant associations in the adjusted model. This suggests that the relationships seen in the binary analysis might be influenced by confounding variables or interactions between the predictors (Table 5).

**Table 5:** Logistic regression of selected factors affecting knowledge of the parents on first aid management of choking child

		Knowledge		Bivariate			Multivariate		
Variable	Categories	Not sufficient	Sufficient	OR	95% CI	p value	OR	95% CI	p value
Gender	Male	42	40	1			1		
	Female	218	125	0.602	0.370–0.978	0.041	0.664	0.394–1.120	0.125
Age (Years)	20 29	59	27	1			1		
	30 39	156	101	1.797	0.957–3.373	0.068	1.661	0.849–3.252	0.138
	40 49	45	37	1.270	0.769–2.098	0.351	1.243	0.736–2.099	0.416
Level of education	Grade 1–5	15	5	1			1		
	upto Ordinary Level	141	72	2.400	0.677–8.505	0.175	2.565	0.712 - 9.242	0.150
	upto Advanced Level	89	76	1.567	0.697–3.523	0.241	1.570	0.690–3.573	0.282
	Degree	15	12	0.937	0.413–2.124	0.876	0.945	0.413–2.163	0.893
Previous first aid	Yes	41	39	1			1		
Training	No	219	126	0.605	0.370 - 0.987	0.044	0.750	0.448–1.253	0.272

## Discussion

Choking refers to the mechanical obstruction of the airways by food or foreign objects, leading to an interruption of breathing. Choking injuries are a substantial cause of morbidity and mortality in children, both at the global and local levels. Choking is the leading cause of infantile death and the fourth most common cause of mortality among preschoole [12]. Results of the study on unnatural childhood deaths presented to the North Colombo teaching hospital in Sri Lanka show seven aspiration-related deaths (18%) involving choking infants [13]. Children under one year of age had the highest choking rates, with children under three accounting for over 75% of choking occurrences [14]. Even though choking injuries are more common in younger children, they have been observed up to 14 years of age [15]. All people, including those outside of the health field should have at least a basic awareness of first aid for choking victims due to the high prevalence of choking and the frequency and rapidity with which choking results in unconsciousness and death. By raising bystander awareness, we can stop most of the fatal choking episodes especially among small children who are typically in adult company [14].

Most choking incidents occur at home during mealtimes and play when parents are the primary supervising adults present. Unlike other medical emergencies, choking demands immediate on-site intervention within minutes to prevent hypoxic brain injury or death. The narrow therapeutic window (approximately 4–6 min before irreversible brain damage) makes parental response capabilities potentially lifesaving. Emergency medical services typically cannot arrive quickly enough to provide the initial critical interventions of back blows and abdominal thrusts needed to dislodge an obstruction, and healthcare professionals rarely witness the actual choking events. Parents therefore occupy an irreplaceable position as the first and most time-critical link in the chain of survival for choking children. This reality necessitates that parents possess both theoretical knowledge and practical competence to execute life-saving techniques under stress. Unlike other aspects of pediatric healthcare choking management fundamentally depends on immediate caregiver action, making parental preparedness a public health priority that warrants systematic evaluation and targeted educational interventions.

This study revealed that none of the participants answered all questions correctly. Only 38.8% achieved a score greater than 14 out of 28 (i.e., > 50%) and were considered knowledgeable, with just 3.3% scoring above 75%. This points towards a generally poor knowledge regarding choking first aid among Sri Lankan parents where the findings align closely with other studies such as among kindergarten teachers in Ethiopia (37%) [1] and Saudi adults in the Eastern Province (43.3%) [16]. The knowledge findings of this study are considerably better than the study in District General Hospital, Kalutara, in which 88.3% of the participants scored below 50% in their knowledge score for choking first aid [11]. The insufficient knowledge of first aid for choking in children among many caregivers is attributed to several interrelated factors. Sri Lanka does not have first aid training in its educational curriculum as a course which is mandatory to train which might be a reason for the low scores in the knowledge assessment. A significant barrier is the underestimation of the risk associated with choking incidents is many parents do not perceive it as a critical concern, leading to a lack of proactive learning. Additionally, there is a notable deficiency in information dissemination from healthcare professionals, resulting in caregivers being unaware of proper first aid techniques. Fear of causing harm or legal repercussions further deters individuals from intervening during choking emergencies. Moreover, limited access to structured training programs and educational resources contributes to this knowledge gap. Addressing these issues through targeted educational initiatives and improved communication from healthcare providers is essential to enhance caregivers’ preparedness in managing pediatric choking incidents effectively [17].

The limited awareness of choking management among parents in Sri Lanka can be attributed to several interrelated factors. First, there is a notable lack of structured, community-based first aid training programs specifically addressing pediatric emergencies, including choking. Public health education in Sri Lanka often focuses on communicable diseases and nutrition, with insufficient emphasis on emergency response training for caregivers. Moreover, choking prevention and management are not routinely integrated into antenatal or postnatal care education, leaving many parents unprepared for such emergencies.

Healthcare providers should incorporate comprehensive choking education into existing maternal and child health programs, emphasizing both prevention and management strategies. Prevention-focused education should include; age-appropriate food preparation: cutting foods into small, manageable pieces; avoiding high-risk foods (whole grapes, nuts, hard candies) for children under 4 years; modifying food textures based on developmental stage, environmental safety measures: ensuring seated eating positions; eliminating distractions during meals (no play, running, or talking while eating); maintaining supervision during mealtimes, toy and small object safety: selecting age-appropriate toys that meet safety standards; keeping small objects (coins, buttons, small toy parts) out of reach; regularly inspecting toys for loose or broken parts and Sri Lanka-specific hazards: educating parents about locally relevant choking risks including traditional sweets (kokis, aluwa), small fruits (rambutan, weralu) commonly given to children, and cultural feeding practices that may increase risk [18].

Socioeconomic disparities and varying levels of health literacy may further contribute to knowledge gaps, particularly in rural and underserved communities. Cultural norms that rely heavily on informal caregiving and traditional practices may also limit parents’ exposure to evidence-based first aid practices [19]. Collectively, these factors highlight the urgent need for targeted interventions and policy level integration of pediatric emergency care education within existing maternal and child health programs.

A notable 80.7% were mothers, indicating that mothers are more commonly the primary caregivers in hospital settings in Sri Lanka. Generally, mothers play a greater role in taking care of their children in Asian cultures. Gender was also significant in bivariate analysis, with males demonstrating higher knowledge levels compared to females. Men often occupy roles in sectors such as security, emergency services, and certain industrial settings where first aid training is mandatory which increases their exposure to first aid education, including choking management techniques. A study in Hail City found that male school teachers had higher knowledge regarding the management of choking emergencies, attributed to their professional training requirements [20]. Women, traditionally seen as primary caregivers, are more involved in managing domestic health emergencies which provides them with practical experience in handling situations like burns and poisoning. Cultural norms may limit women’s participation in formal training programs, affecting their knowledge levels. Conversely, men may have more opportunities to attend such programs through their workplaces or community initiatives.

In this study, 18.8% of participants received prior first aid training. This was similar to Saudi Arabia findings (18.1%) [16] but much lower than reports from Ethiopia (45.5%) [1] and South India (47.3%) [21]. In contrast, a study in Spain reported that 60.4% of study participants had prior training [22]. Kindergarten teachers in Ethiopia with prior first aid training were about three times more knowledgeable than those who did not have it [1]. These studies show that prior training is strongly associated with better first aid knowledge and safer practice, where a significant relationship between previous training and higher knowledge scores was noted in the bivariate analysis.

Despite 87.8% having heard of first aid in choking, only 42.9% scored above 50%. Interestingly, while 79.1% of participants in the study claimed to know how to respond to a choking incident, only 43.1% of them demonstrated adequate knowledge. This gap between perceived and actual competence was found to recur among other international studies as well. For instance, a study in Riyadh showed 73.8% of school teachers claimed proficiency in first aid, but only 38.9% were confident in their skills [23]. In contrast, only 47.8% in Ethiopia and 24% in Poland expressed confidence in their knowledge [1, 24].

When analyzing sources of first aid knowledge, 53.1% cited healthcare professionals, followed by media, family, first aid institutions, school education and other sources. This differs from findings in Kalutara, where family or friends (45.6%), media (30.1%), and school (13.6%) were the main sources, and only 5.8% received information from healthcare professionals [11]. While this reflects some degree of health sector involvement, it also highlights the need for curriculum-based training. Other studies, such as those in Riyadh, report heavy reliance on social media as a source of knowledge [23].

In terms of symptom recognition, 78.1% did not identify the universal sign of choking as clutching the throat, similar to the 68.7% in the Kalutara study [11]. In contrast 53.6% of participated teachers in an Ethiopian study were knowledgeable about the universal sign of choking [1]. Many participants failed to distinguish between complete and partial airway obstruction. Only two participants correctly identified the symptoms of partial obstruction. In a similar study conducted in Ethiopia, 43.8% of the participants had not identified the inability to produce sound or cough as a symptom of complete airway obstruction and 73.7% could not identify symptoms of partial airway obstruction correctly [1]. This shows that majority of the population is unaware of the existence of two types of airway obstruction and is unable to differentiate between symptoms. This is a critical deficiency, as early recognition forms the cornerstone of effective intervention. Without adequate education, caregivers may fail to realize that effective coughing is the body’s natural way of clearing an airway obstruction, and that interrupting this process may cause harm [3].

Encouragingly, many participants could identify risk factors such as eating while playing or talking or running and putting objects in the mouths, likely due to close parental observation. Similar findings were seen in Ethiopia where participants identified that improper chewing of food, immature molars, running and playing with food in their mouths and the adventurous nature of preschool children are potential risk factors for choking [1]. In terms of choking hazards, participants commonly identified items like rambutan seeds, coins, and toffee, similar to results from Ethiopia, where coins (50.9%) were most commonly identified [1]. Common household foods were least likely to be considered hazardous, perhaps due to their familiarity. Only 10.4% of participants correctly identified three minutes as the golden period for initiating first aid management in choking. Guidelines prioritize immediate recognition and action over adherence to specific time limits. Majority (74.8%) responded as two minutes. This misperception may stem from associating choking with urgency, leading many to pick the shortest time available.

In the present study, majority of parents correctly identified back blows as the first step in managing choking. However, only 27.6% knew the correct number of back blows (five), and small number of participants (4.5%) identified abdominal thrusts as the next appropriate step when initial attempts fail. Similarly poor awareness of correct sequence has been observed in other studies, where less than 50% of Saudi adults answered practice-related questions correctly [25]. This could be due to ancestrally carried first aid procedures differing in different ethnicities and geographical regions. These incorrect measures cause more harm to the children and delay proper management as well.

In cases of partial airway obstruction with coughing, more than half incorrectly opted for back blows, while only 19.8% correctly chose to encourage coughing. 23.3% of participants wanted to give a glass of water. This is similar to findings in Kalutara, Sri Lanka where 20.4% wanted to encourage the patient to cough [11]. Several factors contribute to this deficiency. Firstly, there is a prevalent misconception that any choking incident necessitates immediate physical intervention, such as back blows or abdominal thrusts, regardless of the child’s ability to cough which may exacerbate the situation. Secondly, many individuals lack formal training in first aid, resulting in uncertainty about the correct procedures to follow during a choking episode.

In scenarios of unconscious choking, 35% initiated cardiopulmonary resuscitation (CPR) without clearing the airway, 27.3% took the child to the hospital, and another 27.3% did not know what to do after choking. These results lead to confusion about priorities in emergency management. A similar study done in Kalutara, Sri Lanka shows that only 1.9% of mothers would go for CPR [11]. This further adds to the possibility that most of the general population lack proper knowledge or confidence in their knowledge of first aid in choking. This results in them skipping any first aid attempts as it may prolong the time to encounter a doctor.

Regarding choking in infants, more than half correctly chose back blows as the first step. This might be due to postpartum education and strong maternal and child health programs included in public health education in Sri Lanka. This technique is designed to create sufficient pressure and vibration to dislodge the obstructing object from the infant’s airway [26].

Even though this study hypothesized that age, gender, level of education and previous first aid training would significantly associate with knowledge regarding choking management, age and educational level did not show significant associations in the binary analysis. However, in multivariate logistic regression, none of these factors remained statistically significant suggesting that the relationships seen in the binary analysis might be influenced by confounding variables or interactions between the predictors. Interestingly, in contrast to some studies from Saudi Arabia [25] and Ethiopia [1] that found significant associations between higher education or older age with better knowledge. This may suggest that access to training and sources of knowledge, rather than socio-demographic factors, may be the more crucial factor in our setting.

Male parents demonstrated slightly higher knowledge scores, though this association was not statistically significant in adjusted analysis (OR = 0.664, *p* = 0.125). This finding likely reflects structural inequalities in information access rather than inherent differences. In Sri Lanka, men have greater exposure to first aid training through occupational safety requirements in male-dominated sectors (construction, manufacturing, security, transportation), while the maternal and child health system—despite excellent antenatal coverage (> 95%)—predominantly targets mothers through MOH clinics and public health midwife visits scheduled during working hours.

The Child Health Development Record (CHDR), universally distributed to mothers, contains limited choking management content [18]. Traditional gender roles position mothers as primary caregivers responsible for feeding and supervision, yet they receive inadequate formal training in choking prevention and management. Conversely, fathers who may have acquired workplace-based first aid knowledge often have limited involvement in routine childcare activities where choking risks are highest. This misalignment between caregiver roles and knowledge distribution represents a critical gap in Sri Lanka’s healthcare system. These findings emphasize the need for universal, family-centered choking education rather than gender-targeted approaches.

These results reinforce the importance of integrating first aid education, especially in schools and community health programs. Countries with mandatory first aid training, like Spain, show significantly higher awareness, suggesting that policy-level changes in Sri Lanka’s education system could greatly enhance first aid competencies among the general population.

There are several limitations in this study. This single-center study at a tertiary care facility (Sirimavo Bandaranaike Specialized Children’s Hospital, Peradeniya) limits generalizability to other healthcare settings, geographic regions, and socioeconomic contexts. The exclusive inclusion of parents of hospitalized children introduces selection bias, as this population may experience higher stress levels, more severe childhood illnesses, and different healthcare-seeking behaviors compared to parents in primary care or community settings. Parents unable to access tertiary care, those from remote areas, or those with work/childcare constraints preventing hospital visits are underrepresented. Findings may not generalize to parents of children with mild illnesses, outpatient conditions, or those in different healthcare facilities across Sri Lanka.

The interviewer-administered questionnaire introduces potential variability in participant comprehension despite standardized training, written protocols, and language-matched data collectors. Self-reported responses are subject to recall bias, and concurrent child illness during data collection may have compromised parental attention and response quality. Critically, questionnaire-based assessment cannot accurately reflect practical competency in real choking emergencies, as theoretical knowledge does not necessarily translate to effective skill execution under stress. Limited comparable research in Sri Lanka restricted benchmarking opportunities. However, the large sample size, structured assessment tool, adequate response time, and ethnic diversity strengthen validity.

## Conclusion

Knowledge of parents regarding identification of symptoms and signs of choking and provision of first aid for a choking child is insufficient. Main sources of information regarding choking first aid were health care professionals and media. This study emphasizes the need for interventions such as parental education and proper first aid training to improve the parents’ knowledge and skills of choking first aid in order to reduce morbidity and mortality. Further study on large scale should be conducted to generalize findings over the whole population including both urban and rural areas. Addressing the issue relate to disparity in first aid knowledge between males and females requires targeted educational programs that consider occupational roles, societal norms, and access to training. By promoting inclusive and comprehensive first aid education, we can enhance emergency response capabilities across all demographics thereby protecting younger generation.

Ultimately, this research serves as a critical foundation for evidence-based public health interventions that can save lives. Investment in this area of research is not merely academic—it is a public health imperative that has the potential to prevent tragic childhood deaths and reduce the burden of preventable injuries on healthcare systems and families alike.

## Data Availability

The datasets used and/or analyzed during the current study are available from the corresponding author on reasonable request.

## Abbreviations

CPR: Cardiopulmonary resuscitation
